# Responding to Communicable Diseases in Internationally Mobile Populations at Points of Entry and along Porous Borders, Nigeria, Benin, and Togo

**DOI:** 10.3201/eid2313.170520

**Published:** 2017-12

**Authors:** Rebecca D. Merrill, Kimberly Rogers, Sarah Ward, Olubumni Ojo, Clement Glele Kakaī, Tamekloe Tsidi Agbeko, Hassan Garba, Amanda MacGurn, Marydale Oppert, Idrissa Kone, Olutola Bamsa, Dana Schneider, Clive Brown

**Affiliations:** Centers for Disease Control and Prevention, Atlanta, Georgia, USA (R.D. Merrill, K. Rogers, S. Ward, A. MacGurn, M. Oppert, D. Schneider, C. Brown);; Federal Ministry of Health, Abuja, Nigeria (O. Ojo, H. Garba); Ministry of Health, Cotonou, Benin (C.G. Kakaī);; Ministry of Health, Lome, Togo (T.T. Agbeko);; Abidjan Lagos Corridor Organization, Cotonou (I. Kone);; Pro-Health International, Abuja (O. Bamsa)

**Keywords:** border crossing, global health, global health security, public health surveillance, Nigeria, Benin, Togo

## Abstract

Recent multinational disease outbreaks demonstrate the risk of disease spreading globally before public health systems can respond to an event. To ensure global health security, countries need robust multisectoral systems to rapidly detect and respond to domestic or imported communicable diseases. The US Centers for Disease Control and Prevention International Border Team works with the governments of Nigeria, Togo, and Benin, along with Pro-Health International and the Abidjan-Lagos Corridor Organization, to build sustainable International Health Regulations capacities at points of entry (POEs) and along border regions. Together, we strengthen comprehensive national and regional border health systems by developing public health emergency response plans for POEs, conducting qualitative assessments of public health preparedness and response capacities at ground crossings, integrating internationally mobile populations into national health surveillance systems, and formalizing cross-border public health coordination. Achieving comprehensive national and regional border health capacity, which advances overall global health security, necessitates multisectoral dedication to the aforementioned components.

The consequences of insufficient national and regional public health capacities at points of entry (POEs), such as established airports, seaports, or ground crossings, in border regions and among internationally mobile populations became apparent during the 2014–2016 West Africa Ebola epidemic. Within weeks of the first Ebola case in a remote area of Guinea, the epidemic had inconspicuously spread across land borders to Liberia and Sierra Leone (*1*,*2*). A limited number of cases spread over land to Senegal and Mali and through air travel to Nigeria, Spain, and the United States (*3*–*5*). Throughout the almost 2-year epidemic, common local and long-distance international human movement between highly connected communities increased the geographic impact of disease.

National public health systems are designed to detect communicable diseases among established communities and healthcare infrastructure and to respond to minimize their domestic spread. Economic, linguistic, familial, health-seeking, and other factors influence the complexity of cross-border networks. The associated formal and informal international movement challenges national systems’ capacities to detect public health events among these mobile populations (*6*,*7*). Border health strategies minimizing the risk of importation and exportation of disease through POEs, as well as across porous land borders, are not a common feature of national surveillance systems.

In 2005, all World Health Organization (WHO) signatory member states renewed their commitment to addressing the elevated health risks of our increasingly interconnected world by adopting the revised International Health Regulations 2005 (IHR 2005) (*8*). These regulations define legally binding requirements to mitigate the international spread of disease, including required public health capacities at POEs and detection and response collaboration between neighboring and regional countries. Under the IHR 2005, member states are responsible for designating the airports, seaports, and, where justified for public health reasons, ground crossings that must meet POE core capacity requirements defined in the IHR Annex 1 (*8*). Many countries have not yet met IHR 2005 obligations for designated POEs, leaving them particularly vulnerable to possible importation or exportation of communicable diseases (*9*,*10*).

The US Centers for Disease Control and Prevention (CDC) Division of Global Migration and Quarantine (DGMQ), part of the National Center for Emerging and Zoonotic Infectious Diseases, oversees the achievement and maintenance of IHR 2005 core capacities at US POEs. Given this domestic experience, DGMQ began responding to requests for technical assistance from Guinea, Liberia, Sierra Leone, and other regional countries in August 2014 to initiate and strengthen border health measures, primarily exit screening at international airports (*11*). These measures helped Ebola-affected countries meet WHO recommendations, thereby enabling at least some commercial air carriers to continue servicing these countries and providing a vital channel for provision of supplies and response personnel (*12*).

As the number of Ebola cases declined, DGMQ evolved its strategy in the region from outbreak response to longer-term border health capacity building under the premise that effective border health strategies before and during a public health event can help reduce the risk of exporting or importing a communicable disease. Border health strategies could potentially obviate the need for unaffected countries to implement costly entry-screening measures for persons returning from affected countries, as many Western countries did during the Ebola epidemic (*12*,*13*). In this article, we describe a set of border health system strengthening strategies, along with successes and lessons learned from integrating those strategies through partnerships with Nigeria, Benin, and Togo. These countries are highlighted because of their contributions to enhanced global health security through their substantial progress with implementing a comprehensive border health approach.

## Strategies

DGMQ created the International Border Team (IBT), which, with funding from the Global Health Security Agenda, established formal partnerships with 10 countries (Benin, Côte d’Ivoire, Ghana, Guinea, Guinea-Bissau, Liberia, Nigeria, Senegal, Sierra Leone, and Togo) to advance a comprehensive border health strategy (*14*). In this article, we describe in detail the development of each component in this strategy: 1) operational IHR 2005–compliant public health emergency response plans (PHERPs) and supporting standard operating procedures (SOPs) at nationally prioritized POEs; 2) plans for allocating resources to strengthen detection, notification, and referral procedures for prioritized geographic areas and POEs at highest risk for importation or exportation of a high-consequence communicable disease owing to population connectivity and international travel patterns; and 3) timely cross-border and regional public health data sharing, coordination, and collaboration to detect and respond to communicable disease.

### Border Health Strategy 1—Developing POE-Specific PHERPs and SOPs

The IHR 2005 require designated POE to demonstrate capacity for “appropriate public health emergency response by establishing and maintaining a public health emergency contingency plan” (*8*). At many POEs, individual agencies often know appropriate procedures to take during a public health event, yet the procedures are not documented or shared. In the absence of an agreed-upon plan, stakeholders risk gaps or redundancies in communication, surveillance, and response efforts, consequently increasing the risk of an uncoordinated and delayed response. Public health response plans and SOPs are beneficial at IHR-designated POE, as well as at smaller, less-resourced POEs.

A POE PHERP with accompanying SOPs is a multiagency coordination plan that describes procedures to prevent the introduction and transmission of suspected communicable diseases through that POE during routine and response operations. Having the SOPs in writing—available, trained on, and exercised—ensures a timely and coordinated response with all involved sectors. In the airport context, public health, civil aviation, airport authorities, safety and security agencies, airlines, medical and ambulance services, police, and other agencies that have a role in implementing the PHERP are all critical participants in developing, finalizing, exercising, and operationalizing the plan.

The International Civil Aviation Organization (ICAO), the UN specialized agency that ensures that member states’ civil aviation operations and regulations conform to global norms, has developed aviation sector guidelines in accordance with IHR 2005, including those for the development of public health emergency contingency plans at airports (*15*). When developing an airport plan, partners must reconcile ICAO guidance with multiple other global guidance documents as well as other key airport and country planning documents, such as the Aerodrome Emergency Plan, the National Civil Aviation Plan, and the National Public Health Plan. To facilitate the PHERP development process, IBT created a template document, consolidating the WHO Guide for Public Health Emergency Contingency Planning at Designated Points of Entry (*16*) and the ICAO Template for a National Aviation Public Health Emergency Preparedness Plan (*17*). IBT also documented the methodology to create a PHERP through a core planning team, and incorporated lessons learned from DGMQ’s experience in developing communicable disease response plans in the United States. Partner countries have also applied the PHERP development process to seaports and ground crossings.

### Border Health Strategy 2—Establishing Priorities for Capacity Building at Identified POE and Border Regions

Nations often have insufficient financial and personnel resources to build robust border health capacity at all POEs and along entire international borders. To address these challenges, nations can strengthen border health by allocating resources to select POEs and border areas, prioritized by public health risk of importation or exportation of communicable disease, among other considerations. IBT has developed a low-resource field method to gather information from national, subnational, and local stakeholders and community members to characterize population mobility patterns and strength of proximal and distant intercommunity connectivity. This method consists of key informant and focus group discussion guides that a facilitator uses in conjunction with maps of the relevant geographic areas to guide participants through describing the characteristics of those who move into, through, between, and out of identified areas with population movement and connectivity patterns that may increase the impact of a public health event. Nations can use the information, summarized in reports and on maps, to inform their understanding of areas, including POEs, at disproportionately higher public health risk of importation or exportation of communicable disease based on human movement (*18*–*20*).

The WHO IHR 2005 core capacities self-assessment tool enables nations to quantitatively measure current IHR capacities at POEs (*21*). However, the IHR self-assessment tool was developed to evaluate capacities at designated POEs, often international airports, with established infrastructure and resources, and is not as applicable to lower-resource POEs, such as many ground crossings, especially those that are far from urban centers. Further, although the IHR self-assessment tool reserves space to record comments for each question, tool implementation and results analysis focus on the quantitative results. In 2015, IBT developed the Border Health Capacity Discussion Guide (BHCDG) and piloted it in 5 West Africa countries (*22*). The BHCDG complements the IHR self-assessment tool by gathering qualitative information from national, subnational, and border area stakeholders on border health capacities, where infrastructure may not be robust. Nations can use the BHCDG alone or with the IHR self-assessment tool to better understand current capacities and develop an action plan to strengthen gaps in detection, notification, and referral procedures. Specifically, the guide facilitates the collection of qualitative information on the following:

- Communication capacity: communication systems, including identified points of contact for ground crossings, to report and receive notifications of public health events and communication efforts to inform travelers and neighboring communities on public health events or interventions
- Information and data systems: border characteristics, including additional, proximal, unofficial ground crossings, traveler volume, purpose of travel; surveillance systems that incorporate health assessments and responses to public health events at ground crossings; and plans and procedures for public health data sharing with cross-border and regional counterparts about events, such as outbreaks and case investigations
- Response and referral systems: public health and medical services available at and/or near ground crossings and coordination with referral health facilities and response plans and training describing how to prepare for, and respond to, public health events at ground crossings.

### Border Health Strategy 3—Timely Cross-Border and Regional Public Health Collaboration

Effective and timely national health surveillance, coupled with communication and coordination with neighboring and regional countries, supports achieving the IHR principle to protect “all people of the world from the international spread of disease” (IHR 2005 Article 3.3 [*8*]). Through border health strategies 1 and 2, nations build public health capacities at designated and prioritized POEs and border areas to better detect and notify public health events among most international travelers. However, persons travel across porous borders outside a POE or may pass through a POE undetected by health screening measures for several reasons, including being asymptomatic while traveling. Border health strategy 3 addresses the development of cross-border relationships that support prompt communication and coordination between neighboring and regional countries to report and respond to communicable disease events with elevated risk of cross-border transmission.

Nations should incorporate all POE, regardless of infrastructure, into their national health surveillance systems as additional peripheral reporting units expected to follow standard, site-appropriate detecting and reporting practices (*23*). For example, after detecting an ill traveler, a POE official could record event information on a standardized surveillance report form and submit that form to the POE’s referral facility or surveillance unit. Where POEs are not staffed, and along borders without identified POEs, nations can provide communities with additional education to empower them to report potential priority communicable diseases following standard procedures.

To support binational and multinational public health collaboration and coordination, nations can develop and disseminate clear national- and local-level plans that, among other objectives, define when and what public health event information to share across a border, and how to maintain coordination with cross-border counterparts. Some of these collaborations exist informally, but without formalized documentation they may not be clearly defined, may be challenging to supervise, and may not reflect the most current policies and priorities. In addition to documenting domestic plans at the national and local levels, nations can work with neighbors to create integrated cross-border communication and response plans. Real-time data sharing and coordination across borders benefit from maintenance of multinational plans and procedures, along with routine communication to ensure that the plans reflect current priorities.

## Successes and Lessons Learned

### Border Health Strategy 1

Port Health Services of the Federal Ministry of Health in Nigeria, with implementation support from Pro-Health International (PHI) and technical guidance from IBT, began developing, operationalizing, and training staff on PHERPs at 2 international airports: Murtala Muhammed International Airport in Lagos, the 5th busiest airport in Africa, and Nnamdi Azikiwe International Airport in Abuja, the 13th busiest (Figure) (*23*). To develop these plans, Port Health Services, Federal Ministry of Health, airport authority, civil aviation, airlines, immigration, customs, and security partners actively participated in a series of PHERP and SOP development workshops facilitated by IBT and PHI.

**Figure:**
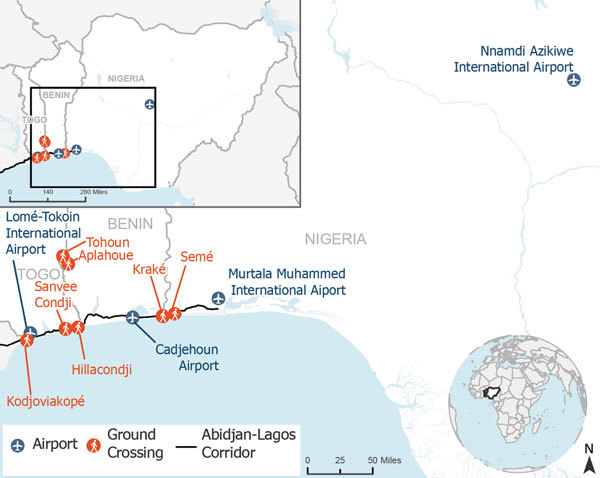
Points of entry within Nigeria, Benin, and Togo targeted for comprehensive border health capacity building through development of public health emergency response plans. Insets show location of enlarged area in West Africa and Africa.

During the introductory PHERP workshops, participants established multiagency core planning teams composed of 8 to 10 persons nominated based on their experience, knowledge, and ability to represent their agencies during the planning process. PHI and Port Health Services, with technical guidance from IBT, facilitated a series of core planning team meetings for Murtala Muhammed International Airport, resulting in a complete PHERP after 10 months. The approved plan now serves as one of the first IHR 2005–compliant PHERPs in West Africa.

The Nnamdi Azikiwe International Airport core planning team, established in March 2016, also finished its PHERP after 11 months of planning. In addition, the core planning team, IBT, and PHI are initiating a new training curriculum and exercise schedule. This training and exercise series is informed by best practices from US CDC quarantine stations and designed to enable responders to execute the PHERP. These tools and workshops can be adapted for use at other types of POE, such as seaports or ground crossings.

### Border Health Strategy 2

In 2016, Togo and Benin, with implementation support from the Abidjan Lagos Corridor Organization (ALCO) and technical guidance from IBT, used IBT field methods to better understand population movement patterns and connectivity related to economic opportunities, healthcare seeking, and cultural festivals, among other factors, with a geographic focus along the international coastal highway—critical because of the high international traveler volume. These countries are working collaboratively, along with IBT and ALCO, to interpret and map the information about crucial points of interest and linguistic, tribal, and other factors associated with the populations that congregate or travel to or through these points. In addition, the countries are using the information to improve national and cross-border surveillance plans including, for example, strengthening preparedness for movement associated with annual celebrations attracting regional visitors. Further, Togo, Benin, and Nigeria are analyzing population mobility and retrospective cholera surveillance data to inform coordinated preparedness and response plans. The countries used this approach to strengthen cross-border coordination during a multinational Lassa fever outbreak in early 2017.

The Benin and Togo ministries of health used the BHCDG following its adoption, in consultation with WHO, ALCO, and IBT, at nationally prioritized ground crossings along the corridor (Kodjoviakopé and Sanvee Condji in Togo and Hillacondji and Kraké in Benin) and a binationally prioritized ground crossing on their shared border (Tohoun and Aplahoue) (Figure). The BHCDG findings gathered from local officials at the POE revealed details about a consistent lack of plans and procedures for responding to public health events, few or no formal mechanisms for collaboration or communication with the neighboring country during a health crisis, and lack of transport and referral mechanisms in place for ill travelers identified at the border. The ministries of health, with technical support from IBT, are implementing an action plan to address the identified areas for improvement using the BHCDG results.

In Nigeria, PHI facilitated BHCDG discussions with personnel at the Semé and Idiroko ground crossings with Benin, the busiest ground crossing in Nigeria. PHI, in collaboration with WHO and the Federal Ministry of Health, adapted the BHCDG to focus on border health human resources, the surveillance system, and binational and regional data sharing—areas not covered in depth by the IHR 2005 self-assessment tool. These discussions occurred 2 weeks after a baseline IHR self-assessment conducted by WHO and national authorities. PHI presented results from the BHCDG discussions to the IHR competent authority and the WHO, who are developing a POE-specific action plan to address gaps identified through the IHR self-assessment and BHCDG activity.

### Border Health Strategy 3

Nigeria’s surveillance system has identified border communities as key components and provides them with tailored training on how to detect and report public health event information. This training, implemented by PHI and the Nigeria Centre for Disease Control, led to improved relationships and communication between border area personnel, health facilities, Local Government Area (Nigeria’s district-level administrative unit) surveillance and Port Health officials, and the national level.

The International Border Team and ALCO, with cosponsoring from the US Agency for International Development (USAID) Benin, facilitated 2 multinational meetings among Nigeria, Benin, Togo, Ghana, and Cote d’Ivoire (which participated in the second meeting only), to formalize cross-border and regional public health data sharing and coordination strategies. Participants included IHR 2005 national focal points, ministry of health legal representatives, national and local public health surveillance leads, national immigration representatives, local port health and quarantine representatives, national and local agricultural and animal health representatives, and the Field Epidemiology Training Program Benin resident advisor. Products from these successful meetings include a draft memorandum of understanding and 7 supporting SOPs and annexes covering the following topics: priority diseases for real-time cross-border reporting; minimum reporting requirements for a cross-border report of a communicable disease; national activities to support cross-border coordination across public health response activation phases; determination of whether a public health event meets criteria for a cross-border report of a communicable disease; determination of whether a public health event meets criteria for responding to a cross-border report of a communicable disease; communication structure for reporting a cross-border event; and communication structure for responding to a cross-border report of a public health event.

In addition to signing the final documents, follow-up steps include consolidating and disseminating cross-border contact information for public health officials working in border districts. Participants noted that they will use the final compendium of jointly produced documents as a training manual for officials working along the borders.

## Discussion

Human mobility is inherently associated with the spread of infectious diseases (*20*,*24*). As transportation networks expand, the speed of travel increases, the volume of passengers and the goods they transport grows, and the potential for the spread of pathogens and their vectors from remote locations to distant countries increases. The Global Health Security Agenda was launched in 2014 to accelerate IHR 2005 implementation to advance global capacity to rapidly detect, respond to, and control public health emergences at their source (*14*). To be maximally effective, a comprehensive global health security agenda must incorporate POEs, border regions, and internationally mobile populations. We have described a set of border health strategies that, when implemented together, are designed to advance national, binational, and regional border health systems. These advanced systems can contribute to improved early detection, effective communication, and timely and adequate response, thereby reducing the risk of international spread of communicable diseases without hindering the free movement of persons and goods.

Border health approaches, for the most part, can leverage tools and strategies in the existing public health and medical systems and infrastructure. The International Border Team’s experience working with partners in Nigeria, Benin, and Togo demonstrates several successes with implementing low-resource methods to strengthen border health capacities. Perhaps the most noteworthy success across all border health strategies was the bringing together of partners to improve multinational and multisectoral collaborations and communication.

Having a written emergency response plan is a key IHR 2005 requirement. Almost complete PHERPs and priority SOPs have been developed for 2 of the highest-volume international airports in West Africa, with others at varying stages of development. Completion, operationalization, and exercising of the PHERPs will help countries meet several of their IHR requirements.

National leaders in each country, along with ALCO and PHI, expressed that the additional information provided by the BHCDG helped them develop a more complete understanding of existing border health capacities and added context to the quantitative results of the IHR 2005 self-assessment. BHCDG information also catalyzed expanding POE-focused capacity-building plans to encompass strengthening communication networks with neighboring countries. The countries plan on using the BHCDG at other priority ground crossings identified, in part, by using information on cross-border population mobility and connectivity.

The challenges experienced to date may be typical of any multisectoral, multinational partnership and were often overcome because of the value placed on the partnerships and in maintaining open dialogue. West Africa has many critical public health challenges; occasional delays in implementation of the comprehensive border health strategy are the result of partners having to respond to competing, higher-priority problems. Achieving consensus on plans and approvals to implement new strategies is time-consuming because of the number of stakeholders who must validate them. Finally, incompatible technology and processes, as well as different languages in neighboring countries, add complexity to information sharing.

Despite the challenges, for resource-limited countries with porous land borders and high cross-border mobility resulting from shared familial, cultural, linguistic, and economic ties, border health security, and therefore health security as a whole, is best achieved by implementing a comprehensive border health strategy involving relevant local, national, and regional sectors. The examples from Nigeria, Benin, and Togo demonstrate that development of a border health system can be successful by including PHERPs for POEs, prioritizing border areas through risk-based assessments using the BHCDG and population mobility mapping, and enhancing timely cross-border surveillance and coordination. Implementing these strategies will help to achieve global health security by supporting countries to prevent the spread of potential health threats across international borders.
